# Whole genome sequencing as a tool to investigate a cluster of seven cases of listeriosis in Austria and Germany, 2011–2013

**DOI:** 10.1111/1469-0691.12638

**Published:** 2014-04-28

**Authors:** D Schmid, F Allerberger, S Huhulescu, A Pietzka, C Amar, S Kleta, R Prager, K Preußel, E Aichinger, A Mellmann, D Raoult

**Affiliations:** 1 German-Austrian Binational Listeria Advisory LaboratoryVienna, Austria; 2 Foodborne Pathogens Reference Services, Public Health EnglandLondon, UK; 3 Federal Institute for Risk AssessmentBerlin, Germany; 4 Robert Koch InstituteBerlin, Germany; 5 Robert Koch InstituteWernigerode, Germany; 6 Baden-Wuerttemberg State Health OfficeStuttgart, Germany; 7 Institute of Hygiene, University of MuensterMuenster, Germany

**Keywords:** Foodborne infections, *Listeria*, listeriosis, outbreaks, typing, whole genome sequencing, zoonoses

## Abstract

A cluster of seven human cases of listeriosis occurred in Austria and in Germany between April 2011 and July 2013. The *Listeria monocytogenes* serovar (SV) 1/2b isolates shared pulsed-field gel electrophoresis (PFGE) and fluorescent amplified fragment length polymorphism (fAFLP) patterns indistinguishable from those from five food producers. The seven human isolates, a control strain with a different PFGE/fAFLP profile and ten food isolates were subjected to whole genome sequencing (WGS) in a blinded fashion. A gene-by-gene comparison (multilocus sequence typing (MLST)+) was performed, and the resulting whole genome allelic profiles were compared using SeqSphere^+^ software version 1.0. On analysis of 2298 genes, the four human outbreak isolates from 2012 to 2013 had different alleles at ≤6 genes, i.e. differed by ≤6 genes from each other; the dendrogram placed these isolates in between five Austrian unaged soft cheese isolates from producer A (≤19-gene difference from the human cluster) and two Austrian ready-to-eat meat isolates from producer B (≤8-gene difference from the human cluster). Both food products appeared on grocery bills prospectively collected by these outbreak cases after hospital discharge. Epidemiological results on food consumption and MLST+ clearly separated the three cases in 2011 from the four 2012–2013 outbreak cases (≥48 different genes). We showed that WGS is capable of discriminating *L. monocytogenes* SV1/2b clones not distinguishable by PFGE and fAFLP. The listeriosis outbreak described clearly underlines the potential of sequence-based typing methods to offer enhanced resolution and comparability of typing systems for public health applications.

## Introduction

The large majority of listeriosis cases (sporadic and outbreak-related) are caused by foodborne transmission, which accounts for 99% of human cases 1,2. In an era when food production and food storage rely heavily on refrigeration, the ability of *Listeria monocytogenes* to grow (albeit slowly) at low temperatures has opened a new ecological niche to a pathogen that previously had only mediocre relevance. Industrialized food manufacturing also constitutes an ecological niche, owing to the ability of *L. monocytogenes* to form biofilms for colonization of surfaces 3. Seeliger even dubbed this zoonosis a ‘man-made disease’ 4.

**Fig 1:**
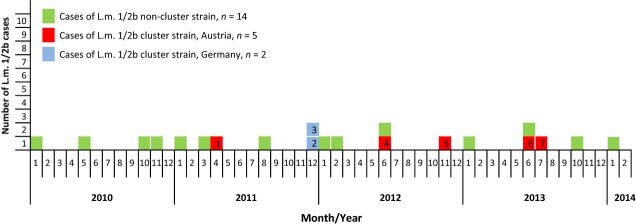
Cases of invasive listeriosis caused by *Listeria monocytogenes* (L.m.)serovar 1/2b by calendar week of diagnosis (Austria, 2010 to February 2014).

In Austria, laboratories have a legal obligation to forward human and food *L. monocytogenes* isolates to the national reference centre; however, the source of the food isolates does not have to be notified to the reference laboratory. On 21 January 2013, the Austrian Food Authority mandated the Austrian Agency for Health and Food Safety to investigate the source and and public health significance of a certain pulsed-field gel electrophoresis (PFGE) clone of *L. monocytogenes* serovar (SV) 1/2b, which, in 2012, had become the dominant *Listeria* strain among food isolates; the cluster strain accounted for 29 of 50 *L. monocytogenes* SV1/2b food isolates received at the National Reference Laboratory during the second half of 2012. At the beginning of the cluster investigation, only two invasive cases of listeriosis with this strain were known. Active case-finding revealed seven human cases of invasive listeriosis caused by *L. monocytogenes* SV1/2b sharing PFGE (*Asc*I, *Apa*I, and *Sma*I) and fluorescent amplified fragment length polymorphism (fAFLP) (*Hha*I and *Hin*dIII) patterns (defined as ‘1/2b cluster strain’) (Fig.1). All cluster cases were female, the median age was 75 years (range: 71–83 years), and two cases had fatal outcomes; between April 2011 and July 2013, five cases (cases 1 and 4–7) occurred in Austria and two cases occurred in Germany (cases 2 and 3). We found isolates from food products of five producers (two in Austria, two in Germany, and one in Romania) that were indistinguishable from the human cluster isolates by PFGE (*Asc*I, *Apa*I, and *Sma*I) and fAFLP (*Hha*I and *Hin*dIII). Epidemiological investigation suggested that cluster cases 4–7 (Austria, 2012 and 2013) were linked to either one of the two Austrian food producers (A and B). For cluster case 1 (Austria, 2011) and cluster cases 2 and 3 (Germany, 2011), the incriminated ready-to eat (RTE) meat products of producer B had been excluded as possible sources by the findings of the epidemiological investigation. To further investigate this listeriosis cluster, we applied whole genome sequencing (WGS) and subsequent gene-by-gene comparison (multilocus sequence typing (MLST)+).

## Materials and Methods

### Microorganisms

A total of 18 *L. monocytogenes* SV1/2b isolates were included in the WGS and subsequent gene-by-gene comparison (MLST+): they originated from the five Austrian cluster cases (AT01, 2011; AT02 and AT03, 2012; AT16 and AT18, 2013), two German cluster cases (AT14 and AT15, 2011), five Austrian unaged soft cheeses from producer A (AT05–AT08 and AT17, 2013), two Austrian RTE meat products from producer B (AT09 and AT10, 2013), one Romanian RTE meat product (AT11, 2012), and two German RTE meat products (AT12 and AT13, 2010). A human isolate (AT04) with a different PFGE/fAFLP profile was included as a control. PFGE and fAFLP were performed as described elsewhere 5–8. Table1 shows the salient features of these 18 isolates.

**Table 1:** Summarized data on 18 *Listeria monocytogenes* serovar 1/2b isolates tested with whole genome sequencing, with all but isolate AT04 (control strain) yielding pulsed-field gel electrophoresis and fluorescent amplified fragment length polymorphism patterns that were indistinguishable from each other

Isolate ID	Source	Details for patients (age in years, sex) and for the implicated food	Country/Year
AT01	Human (case 1)	75, female (fatal outcome)	Austria, 2011
AT02	Human (case 4)	81, female	Austria, 2012
AT03	Human (case 5)	77, female	Austria, 2012
AT04	Human (control strain)	Newborn (epidemiologically unrelated case)	Austria, 2013
AT05	Food	Fresh cheese (sheep) from producer A	Austria, 2013
AT06	Food	Fresh cheese (sheep) from producer A	Austria, 2013
AT07	Food	Fresh cheese (sheep) from producer A	Austria, 2013
AT08	Food	Fresh cheese (goat) from producer A	Austria, 2013
AT09	Food	Sliced ham (pork) from producer B	Austria, 2013
AT10	Food	Pate (pork) from producer B	Austria, 2013
AT11	Food	Frankfurter sausage (turkey) from producer C	Romania, 2012
AT12	Food	Poultry meat (chicken) from producer C	Germany, 2010
AT13	Food	‘Fleischsalat’ (salad with strips of bologna-type sausage) from producer D	Germany, 2010
AT14	Human (case 2)	72, female (fatal outcome)	Germany, 2011
AT15	Human (case 3)	81, female	Germany, 2011
AT16	Human (case 6)	83, female	Austria, 2013
AT17	Food	Fresh cheese (sheep) from producer A	Austria, 2013
AT18	Human (case 7)	71, female	Austria, 2013

### WGS and data analysis

The 18 *Listeria* isolates were subjected to WGS in a blinded fashion. A single colony of a fresh culture was incubated overnight in brain–heart infusion broth prior to DNA extraction. One microlitre of this culture was subsequently subjected to DNA extraction with the Qiagen MagAttract HMW DNA kit (Qiagen, Hilden, Germany), according to the manufacturer's recommendations. Sequencing libraries containing 1 ng of DNA were prepared with Nextera XT chemistry (Illumina, San Diego, CA, USA) for a 75-bp, 150-bp or 250-bp paired-end sequencing run on an Illumina MiSeq sequencer, according to the manufacturer's recommendations (Illumina), aiming for a minimum coverage of 75-fold 9. After quality trimming with the default parameters of the CLC Genomic Workbench software version 6 (CLC bio, Arhus, Denmark), the sequencing reads were assembled with the CLC Genomic Workbench *de novo* assembler with default parameters, with the exception of ‘length fraction value = 0.8’ (CLC bio). Gene sequences for subsequent analyses were extracted from contigs with Ridom SeqSphere^+^ software version 1.0 (Ridom, Muenster, Germany). For the gene-by-gene analysis, i.e. MLST+, as described earlier, we initially compiled a list of genes that were present in all strains analysed 10. This list, comprising 2,298 genes used for *L. monocytogenes* MLST+ typing, is accessible under http://www.ages.at/ages/gesundheit/mensch/listeriose/gene-by-gene-typing-of-l-monocytogenes-isolates/. The designation of the analysed genes was based on *L. monocytogenes* strain EGD-e (GenBank accession no. NC_003210). For phylogenetic analysis, the defined cgMLST sequences, i.e. the 2298 gene sequences, were extracted from each assembly and assessed for quality, i.e. the absence of premature stop codons and ambiguous nucleotides, and a minimum sequence coverage of ten-fold or more over the whole gene with a minimum substitution frequency in reads of 75%. If genes fulfilled all of these quality criteria, their complete sequence was analysed in comparison with *L. monocytogenes* strain EGD-e, and a numerical allele type was assigned by SeqSphere^+^. For illustration of the phylogenetic relationship of the 18 *L. monocytogenes* isolates, the combination of all alleles in each strain formed an allelic profile, i.e. the typing result, which was used for subsequent construction of a minimum-spanning tree with SeqSphere^+^ software (Ridom). The whole genome sequence reads have been deposited at the European Nucleotide Archive (study accession no. PRJEB5481), and detailed WGS characteristics are shown in Table2.

**Table 2:** Detailed whole genome sequencing results of the 18 *Listeria monocytogenes* serovar 1/2b genomes and European Nucleotide Archive (ENA) sample accession numbers (study accession no. PRJEB5481)

Isolate ID	No. of assembled reads	No. of consensus sequence nucleotides (bp)	No. of contigs	Average coverage	No. of deduced genes^a^	ENA sample accession no.
AT01	1 113 738	3 011 773	49	55.0	2406	ERS406789
AT02	1 354 880	3 013 172	44	66.8	2429	ERS407319
AT03	1 524 321	3 012 460	36	75.5	2436	ERS407320
AT04	1 612 866	3 025 964	44	79.4	2427	ERS407321
AT05	2 227 840	3 014 120	39	108.7	2430	ERS421601
AT06	2 357 928	3 012 372	44	115.9	2428	ERS427372
AT07	2 127 139	3 014 249	41	105.1	2439	ERS427979
AT08	1 511 647	3 013 045	41	74.5	2434	ERS427980
AT09	1 245 078	3 012 059	49	60.7	2425	ERS428001
AT10	5 706 271	3 015 193	51	152.6	2444	ERS408418
AT11	1 474 303	3 008 842	41	73.0	2433	ERS428003
AT12	5 893 062	2 931 547	75	159.2	2447	ERS421884
AT13	3 398 658	2 986 020	42	97.0	2445	ERS428004
AT14	998 085	3 012 269	54	48.8	2393	ERS428005
AT15	5 772 962	3 012 616	47	159.3	2445	ERS428009
AT16	974 292	3 011 808	47	48.2	2415	ERS428006
AT17	2 222 068	3 012 212	47	109.9	2427	ERS428007
AT18	1 726 262	3 028 709	74	136.3	2426	ERS428008

a In a pairwise comparison with *L. monocytogenes* strain EGD-e (GenBank accession no. NC_003210).

## Results

**Fig 2:**
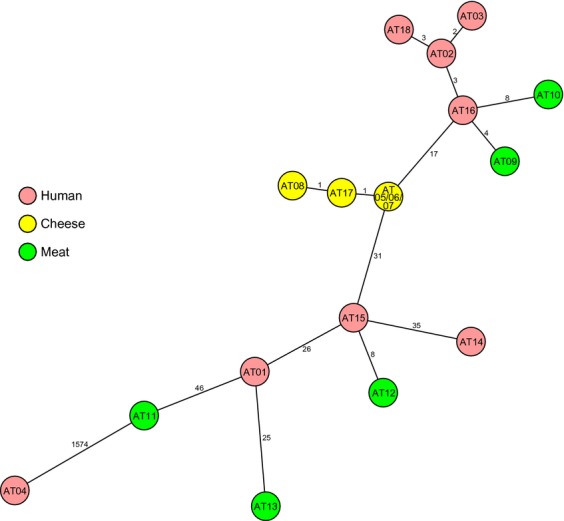
Phylogenetic relationships of 18 *Listeria monocytogenes* serovar 1/2b isolates from different sources based on whole genome sequencing. The minimum spanning tree is based on allelic profiles of 2998 genes present in all strains investigated. Each circle represents a given allelic profile, and contains the isolate's name as given in Materials and Methods. The different sample origin (human, cheese, and meat) is distinguished by colours of the circles. The numbers on the connecting lines illustrate the number of differing alleles in a pairwise comparison.

After quality control of the WGS data, 2298 genes were finally analysed: isolates AT02, AT03, AT16 and AT18 from Austrian cluster cases 4–7 had different alleles at ≤6 genes, i.e. differed by ≤6 genes from each other (defined as outbreak cases); the dendrogram placed these isolates in between the Austrian unaged soft cheese isolates (AT05–AT08 and AT17) from producer A (≤19-gene difference from the human cluster) and the RTE meat isolates (AT09 and AT10) from producer B (≤8-gene difference from the human cluster) (Fig.2). In contrast to the German and Romanian food products, these two Austrian food products, which harboured *L. monocytogenes* at <30 CFU/g, appeared on grocery bills collected from the outbreak cases (cases 4–7) after hospital discharge. MLST+ clearly separated the isolates from Austrian cluster case 1 (AT01) and German cluster cases 2 and 3 (AT14 and AT15) from the outbreak cases' isolates (≥48 different genes). The two German meat isolates (AT12 and AT13) and the Romanian meat isolate (AT11) showed a ≥56-gene difference from the outbreak cases' isolates. The control SV1/2b strain (AT04) differed from the isolates with indistinguishable PFGE/fAFLP patterns by 1574 genes.

## Discussion

*L. monocytogenes* is a particularly important cause of illness, and is mainly found in foods that are packaged and prepared commercially, rather than those prepared in the home 11. During recent decades, consumer lifestyles have changed, with less time available for food preparation, and higher consumption of RTE and takeaway foods. Changes in food production and technology have led to the production of foods with a long shelf-life that are typical ‘*Listeria* risk foods’, because the psychrotrophic bacteria have time to multiply, and the food does not undergo a listericidal process such as cooking before consumption. Swaminathan named the high degree of centralization and consolidation of food production and processing, the increased use of refrigerators as the primary means of preserving food and the above-mentioned changes in food consumption habits (increased consumer demand for convenient food) as the main factors increasing the incidence of listeriosis 12.

Epidemiological investigation suggested that four of the five Austrian cluster cases of *L. monocytogenes* SV1/2b (cases 4–7; 2012 and 2013) were linked with either one of the two microbiologically related Austrian food producers; food products from both producers appeared on grocery bills collected from these cases after hospital discharge. Already in a previous outbreak, all surviving Austrian outbreak cases had been asked to collect grocery receipts for the 3 weeks after they were discharged from hospital, to enable the collection of information on routine food consumption behaviour 13,14. This approach successfully revealed ‘Quargel’, a type of acid curd cheese available in different flavours, as the source of the 2009–2010 outbreak, which involved 34 cases of invasive listeriosis (including eight fatalities) 15.

For Austrian cluster case 1 and the German cluster cases (cases 2 and 3), all of which occurred in 2011, the incriminated RTE meat products of producer B had been excluded as possible sources by epidemiological investigation (data not shown). MLST+ was able to discriminate *L. monocytogenes* isolates from these three cluster cases, and these isolates were related according to PFGE and fAFLP. On the basis of these MLST+ findings, cluster cases 1–3 could be excluded from the outbreak, and the meat products from Romania and Germany were ruled out as possible sources of infection. Without MLST+, the three 2011 cases (including two fatalities) would have been incorrectly considered to be outbreak-related, resulting in the false exclusion of RTE meat products of producer B, which were sold neither in Germany nor in the sole grocery store frequented by case 1, as a possible source.

Our investigation suggests that the outbreak cases (cases 4–7; 2012 and 2013) were linked with either one of the two Austrian food producers. However, none of the 34 food samples positive for the SV1/2b cluster strain with quantitative data available yielded *L. monocytogenes* in numbers above 100 CFU/g (data not shown), the legal threshold in the EU.

**Fig 3:**
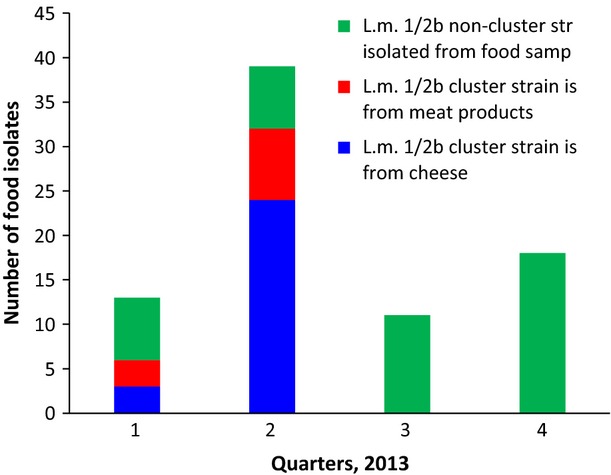
Occurrence of *Listeria monocytogenes* serovar 1/2b in food samples tested in Austria 2013: cluster strain in comparison with non-cluster strains. Outbreak investigation started on 22 January 2013. The producer of the unaged soft cheese, producer A, was first contacted on 26 February, and the producer of the ready-to-eat meat, producer B, on 4 March. Raised awareness and resulting intensification of in-factory sanitation caused the cluster strain to disappear.

We were unable to determine the causative vehicle among two biologically plausible suspects left: unaged soft cheese and shrink-wrapped deli meat. However, raised awareness caused by the outbreak investigation and the resulting intensification of in-factory sanitation stopped the detection of the cluster strain in food samples (Fig.3). The producer of the fresh soft cheese, producer A, was first contacted on 26 February 2013, and the producer of the RTE meat, producer B, on 4 March 2013. At the fresh soft cheese production facility of producer A, routine testing for *L. monocytogenes* of every batch produced (with negative results being a prerequisite for any delivery) was implemented. At the RTE meat production facility of producer B, a meat-slicer that, by intensified environmental surveillance, was identified as a possible source of *L. monocytogenes* contamination was sanitized. To date (February 2014), no further cases caused by the *L. monocytogenes* SV1/2b cluster strain have been reported since 15 July 2013 (hospitalization date of case 7). This shows that outbreak investigation and general public health action can control an outbreak even in the absence of definitive proof of a causative vehicle.

In contrast to the situation in forensic human genetics, where—with the exception of monozygotic twins—indistinguishable DNA fingerprinting patterns prove epidemiological relatedness, in bacteriology even epidemiologically unrelated isolates can yield DNA fingerprinting patterns that are indistinguishable from each other. Explanations for this might be a monomorphic population structure, which is known from other pathogens such as *Escherichia coli* O157:H7, or the limitation of the typing method used 16. As our data show for *L. monocytogenes*, pure chance can yield pairs of epidemiologically unrelated isolates with PFGE and fAFLP patterns that are indistinguishable from each other, and pairs of contemporary isolates of human and of food origin without any causal relationship. On the other hand, the maximum resolution of WGS poses a major challenge in determining meaningful similarity thresholds for grouping of related isolates to provide an appropriate level of discrimination for source attribution. The definition of such thresholds will be a major task for the scientific community and the public health authorities, using well-defined outbreak scenarios of every pathogen species of interest. The applied method has to allow for some genetic diversity between isolates from human and food sources, but only to the degree that it can still be assumed that they originate from the same source 17. WGS and genome analyses have already proven their potential to identify genes that contribute to persistence of *L. monocytogenes* in food production plants and to virulence 18,19. At present, we lack knowledge on the expected rate of change of *L. monocytogenes* in our MLST+ scheme over time, on the expected diversity within outbreak strains, and on the expected diversity between outbreak strains and source isolates. We see a clear need for this diversity information before this MLST+ scheme can be used for source attribution rather than exclusion. However, we have shown that comparative WGS with gene-by-gene-based data analysis (MLST+) is capable of discriminating *L. monocytogenes* SV1/2b clones grouped together by the genotyping methods PFGE (*Asc*I, *Apa*I, and *Sma*I) and fAFLP (*Hha*I and *Hin*dIII). The meeting participants of a recent ECDC technical consultation on harnessing genomics for epidemiological surveillance (Paris, 1–2 October 2013) unanimously agreed that WGS will replace current typing techniques for surveillance and outbreak investigation 20. The listeriosis outbreak described clearly underlines the potential of sequence-based typing methods to offer enhanced resolution and comparability of typing systems for public health applications.

## Author Contributions

D. Schmid, F. Allerberger, C. Amar, and A. Mellmann: wrote the manuscript. D. Schmid, F. Allerberge, S. Huhulescu, A. Pietzka, C. Amar, S. Kleta, R. Prager, E. Aichinger, and A. Mellmann M: performed laboratory or epidemiological investigations.

## Transparency Declaration

The authors declare no conflicts of interest.
